# Supplementary material for: Emotion regulation as a transdiagnostic link between ADHD and depression symptoms: evidence from a network analysis of youth in the ABCD study
Source: Child Adolesc Psychiatry Ment Health. 2025 Oct 21;19:113. doi: 10.1186/s13034-025-00966-6 (PMC12538765; doi:10.1186/s13034-025-00966-6)
Supplement: Supplementary file 1 — Supplementary Material [file 13034_2025_966_MOESM1_ESM.docx]

**Supplementary Information**

**Article:** Emotion Regulation as a Transdiagnostic Link Between ADHD and Depression Symptoms: Evidence from a Network Analysis of Youth in the ABCD Study

**Journal:** Child and Adolescent Psychiatry and Mental Health

**Figure S1**

*Bootstrapped confidence intervals of estimated edge weights.*

******

*Note.* Gray lines indicate confidence intervals around edge weight estimates.

**Figure S2**

*Stability of network centrality measures.*

*Note.* The graphs indicate the average correlation between different proportions of the full sample and the original sample for A. expected influence and strength; and B. bridge expected influence. The CS-coefficient threshold was set a priori to .50, indicating that 50% of sampled cases can be dropped to maintain a correlation of .70 with the full sample with 95% confidence.

**Figure S3**

*Bootstrapped difference tests between expected influence of nodes in the network.*

**

*Note.* Black squares indicate significant differences in expected influence, *p* < .05. Gray squares indicate comparisons that are not significantly different, *p* > .05.

**Figure S4**

*Bootstrapped difference tests between strength centrality values of nodes in the network.*

******

*Note.* Black squares indicate significant differences in strength centrality, *p* < .05. Gray squares indicate comparisons that are not significantly different, *p* > .05.

**Figure S5**

*Bootstrapped difference tests between bridge expected influence values in the network.*

*Note.* Black squares indicate significant differences in bridge expected influence, *p* < .05. Gray squares indicate comparisons that are not significantly different, *p* > .05.

**Table S1**

*Centrality stability coefficients*

| Centrality Index | CS-coefficient |
| --- | --- |
| Strength | .52 |
| Expected Influence | .75 |
| Betweenness | .13 |
| Closeness | .36 |
| Bridge Strength | .52 |
| Bridge Expected Influence | .75 |
| Bridge Betweenness | .21 |
| Bridge Closeness | .36 |

*Note.* CS-coefficient = centrality stability coefficient, indicating the proportion of the sample that can be dropped while maintaining a correlation of .70 with the full sample with 95% confidence. Betweenness, closeness, bridge betweenness, and bridge closeness did not reach the .50 threshold set a priori and were not interpreted in the main text. Bridge strength was also not interpreted because it is influenced by negative edges. Strength, expected influence, and bridge expected influence met the threshold for stability and were interpreted in the main text.

**Table S2**

*Partial Correlations Between Emotion Regulation Subscales and Symptoms of ADHD and Depression.*

| Node | Attuned | Catastrophize | Neg_Sec | Distract |
| --- | --- | --- | --- | --- |
| 1. Finish_things | .06 | 0 | 0 | .08 |
| 2. Concentrate | .01 | 0 | 0 | .01 |
| 3. Hyperactive | 0 | .05 | 0 | 0 |
| 4. Impulsive | .03 | .14 | -.08 | .01 |
| 5. Inattentive | .02 | -.07 | -.01 | .09 |
| 6. Talks_much | -.08 | 0 | .04 | -.04 |
| 7. Too_loud | 0 | .07 | -.04 | 0 |
| 8. Enjoys_little | .08 | .03 | -.03 | 0 |
| 9. Cries_lot | -.10 | .08 | -.05 | 0 |
| 10. Eats_well | .05 | .02 | -.05 | .04 |
| 11. Worthless | .06 | 0 | .08 | .04 |
| 12. Too_guilty | -.03 | 0 | .12 | .03 |
| 13. Overtired | .02 | 0 | 0 | 0 |
| 14. Less_sleep | -.03 | 0 | .07 | 0 |
| 15. More_sleep | .01 | 0 | .08 | -.04 |
| 16. Trouble_sleep | 0 | 0 | -.09 | .03 |
| 17. Underactive | 0 | -.05 | 0 | .03 |
| 18. Unhappy | 0 | .01 | -.01 | .01 |
| 19. Attuned | - | .24 | -.08 | -.06 |
| 20. Catastrophize | .24 | - | .38 | .51 |
| 21. Neg_Sec | -.08 | .38 | - | .13 |
| 22. Distract | -.06 | .51 | .13 | - |

*Note.* 0 = no significant partial correlation. The table displays bootstrapped sample edge weights (partial correlations) for network edges connecting emotion regulation subscales (Attuned, Catastrophize, Negative Secondary Emotions, and Distracted) to other nodes in the network.

We conducted sensitivity analyses of centrality and bridge centrality indices in the full network model to test its robustness when including the two removed DERS-P items in the DERS-P subscale scores, omitting participants with incomplete data (*n* = 4,460), and omitting including parents who reported completing the DERS-P in Spanish (*n* = 9,516). Strength centrality, expected influence, and bridge expected influence estimates remained very similar across all three analyses (see values in Table S2), and the rank order of DERS-P subscales by all three measures remained consistent with the order found in the full sample and with the two DERS-P items removed.

We also estimated a network model with item-level DERS-P data to examine whether any notable differences emerged from the subscale-level model. For both strength and expected influence, items from the Catastrophize subscale related to difficulty remaining in control when upset were the DERS-P items with the highest values: “loses control over his/her behaviors when upset” followed by “out of control when upset” for strength, and “feels out of control when upset” for expected influence. The next-highest expected influence items were tied between “pays attention to how he/she feels” from the Attuned subscale and the “ashamed” item from the Negative Secondary subscale. Interestingly, the Catastrophize items with the highest bridge expected influence in the sample were experiencing “emotions as overwhelming and out of control” and feeling emotions are “overwhelming when upset”. The item from the Distracted subscale with the highest bridge expected influence was “difficulty focusing on other things when upset”.

**Adolescent Self-Reported Depression Symptoms**

Finally, we estimated a model using adolescent self-report of depression symptoms in place of parent-reported depression symptoms at year 4 (ages 12-13). Adolescents self-reported their depression symptoms in the ABCD study using the Brief Problem Monitor (BPM). The BPM contains three depression items but is from the same family of instruments as the parent-report CBCL [1]. The three depression items (“I feel worthless or inferior”, “I feel too guilty”, “I am unhappy, sad, or depressed”) overlap in content with three CBCL depression items. The same pattern of overall strength and expected influence remained among DERS-P subscales when using youth self-reported depression symptoms. Although Catastrophize had the highest bridge expected influence of the four DERS-P subscales (see Figure S6), bootstrapped difference tests found that the difference between Catastrophize and Distracted was not significant, *p* > .05 (see Figure S7), consistent with the parent-report findings in the full text.

Omnibus network comparison tests were consistent with the parent-reported network in the full-text, with some differences in centrality and edge invariance tests. Network comparison tests followed the same analytic strategy as described in the full text and corrected for multiple comparisons. Networks compared by sex found no significant differences in the network invariance test (*p* = .314), the invariant global strength test (*p* = .969). There were no differences in centrality invariance tests of individual network nodes. Networks compared by history of ADHD diagnosis on the K-SADS resulted in a significant network invariance test (*p* = .002) but no significant differences in global strength between the networks (*p* = .455). Follow-up examination of edge invariance tests found four edges that were significantly stronger in the ADHD network than the non-ADHD network: the edge between “hyperactive” and “impulsive” ADHD CBCL symptoms (*p* = .012) and the edges between three pairs of DERS-P subscales (“Attuned” and “Negative Secondary Emotions”; “Catastrophize” and “Distracted”; and “Negative Secondary Emotions” and “Distracted), *p*s range between .012 and .018. Surprisingly, the only significant centrality invariance test found that Negative Secondary Emotions had significantly greater expected influence in the non-ADHD network (*p* = .045).

Networks compared by ADHD polygenic risk score (PRS) higher vs. lower than the median of the sample resulted in a significant network invariance test (*p* < .001) and a significant global strength test (*p* < .001), indicating that the high ADHD PRS network has overall stronger edges than the low ADHD PRS network. Follow-up examination of edge invariance tests found six edges that were significantly stronger in the ADHD network than the non-ADHD network: the edge between Catastrophize and Distracted (*p* = .006) and five edges between pairs of ADHD symptoms (“hyperactive” and “impulsive”; “concentrate” and inattentive”; “impulsive” and “inattentive”; “hyperactive” and “too loud”; “talks much” and “too loud”), all *p*s = .006. Centrality invariance tests found that all seven ADHD symptoms had significantly greater strength and expected influence in the high ADHD PRS network (all *p*s = .003). Interestingly, three of the four DERS-P subscales (with the exception of Distracted) had significantly greater expected influence in the high ADHD PRS network than the low ADHD PRS network, *p*s range from .003 to .005. Catastrophize also had significantly greater strength in the high ADHD PRS network than the low ADHD PRS network (*p* = .013). There were no significant differences between the networks involving depression items.

In sum, the overall pattern of results in both the main network and in exploratory network comparison tests remains consistent when using the three self-reported depression items instead of the parent-reported depression items reported in the full text. These findings support the robustness of the results for both self-report and parent-report of adolescent depression symptoms even in a network with fewer nodes.

**Multiple Imputation and Randomly-Selected Siblings**

We applied multiple imputation by chained equations using the *mice* package version 3.18.0 in R with 20 imputations and a maximum of 10 iterations (Nehler & Schultze, 2025). ADHD CBCL items at baseline, year 1, and year 2 and DERS-SF items at year 3 were imputed. Depression year 4 items were not imputed due to higher levels of missingness that prevented convergence. Auxiliary variables included sex, race/ethnicity, caregiver (i.e., mother, father, etc.), parental marital status, parental employment, parent language preference (i.e., English or Spanish), study site, and family ID. Variables measured at later timepoints could not predict variables measured at earlier timepoints. The quickpred function was used to minimize predictors to those correlated at or above .30 to facilitate convergence and efficiently utilize computational resources. Next, we randomly selected one sibling for each family across each imputation.

Network bridge expected influence (EI) values for DERS subscales for each of the 20 imputations conducted were consistent with findings in the main text: Catastrophize and Distracted were the strongest bridges followed by Negative Secondary Emotions, and Attuned, respectively. Network comparison tests found that networks did not differ by sex in network structure (*p*s ranged from .107 to .766) or global strength (*p*s ranged from .052 to .852). Network comparisons by lifetime ADHD diagnosis on the K-SADS found that networks did not differ in global strength (*p*s ranged from .124 to .911) but that one of the 20 iterations significantly differed in network structure (*p*s ranged from .037 to .286). Network comparisons by ADHD polygenic score (PGS) found that high PGS networks had significantly greater global strength from low PGS networks (all *p*s < .001) and significant differences in network structure (all *p*s < .005). Results using multiply imputed datasets and selecting one sibling per family are consistent with the results reported in the main text. The exception in the K-SADS network comparisons may be in part due to reduced power associated with reductions in sample size and unequal sample sizes across the networks of individuals with and without lifetime history of ADHD.

References

1. Achenbach TM, Rescorla LA. *Manual for the ASEBA School-Age Forms & Profiles.* University of Vermont: Research Center for Children, Youth, and Families.

**Table S3**

*Strength, Expected Influence, and Bridge Expected Influence in Sensitivity Analyses.*

| Node | Strength | EI | Bridge EI |
| --- | --- | --- | --- |
| Youth-Reported Depression Items | | | |
| Attuned | 0.68 | 0.19 | 0.07 |
| Catastrophize | 1.53 | 1.38 | 0.22 |
| Neg_Sec | 0.83 | 0.43 | -0.02 |
| Distract | 0.99 | 0.83 | 0.21 |
| All Items Included | | | |
| Attuned | 0.95 | 0.19 | 0.10 |
| Catastrophize | 1.65 | 1.41 | 0.27 |
| Neg_Sec | 1.26 | 0.43 | -0.00 |
| Distract | 1.09 | 0.86 | 0.31 |
| Complete Cases | | | |
| Attuned | 1.01 | 0.20 | 0.11 |
| Catastrophize | 1.59 | 1.40 | 0.26 |
| Neg_Sec | 1.31 | 0.42 | 0.03 |
| Distract | 1.14 | 0.86 | 0.27 |
| English-Speakers Only | | | |
| Attuned | 0.94 | 0.21 | 0.10 |
| Catastrophize | 1.65 | 1.41 | 0.28 |
| Neg_Sec | 1.26 | 0.45 | -0.01 |
| Distract | 1.09 | 0.89 | 0.33 |
| Item-Level Analyses | | | |
| Subscale: Attuned |  |  |  |
| Pays attention to how he/she feels | 1.65 | 1.23 | -0.01 |
| Clear about his/her feelings | 1.48 | 0.87 | 0.03 |
| Attentive to his/her feelings | 1.26 | 0.84 | 0.09 |
| Cares about what he/she is feeling | 1.78 | 0.88 | -0.04 |
| Knows exactly how he/she is feeling | 1.81 | 1.12 | 0.05 |
| Acknowledges his/her emotions when upset | 1.51 | 0.69 | -0.08 |
| Subscale: Catastrophize |  |  |  |
| Emotions as overwhelming and out of control | 1.71 | 0.72 | 0.14 |
| Can remain in control of his/her behaviors when upset | 1.64 | 0.62 | 0.03 |
| Difficulty controlling his/her behaviors when upset | 1.87 | 1.04 | 0.07 |
| Knows that he/she can find a way to feel better when upset | 1.87 | 0.87 | 0.06 |
| Out of control when upset | 2.35 | 1.16 | 0.09 |
| Emotions feel overwhelming when upset | 1.75 | 1.04 | 0.12 |
| Believes he/she cannot make him/herself feel better when upset. | 1.55 | 1.04 | 0.04 |
| Takes him/her a long time to feel better when upset | 1.45 | 0.97 | 0.01 |
| Loses control over his/her behaviors when upset | 2.61 | 1.08 | -0.05 |
| Feels out of control when upset | 1.74 | 1.25 | 0.01 |
| Believes he/she will remain upset for a long time | 1.97 | 0.98 | -0.06 |
| Subscale: Negative Secondary |  |  |  |
| Angry at self for feeling upset | 1.71 | 0.87 | 0.02 |
| Ashamed with self for feeling upset | 2.10 | 1.23 | 0.05 |
| Embarrassed for feeling upset | 1.66 | 0.86 | -0.03 |
| Feels very bad about self when upset | 1.53 | 1.15 | 0.10 |
| Guilty for feeling upset | 1.71 | 0.71 | 0.01 |
| Irritated with self for feeling upset | 1.89 | 1.05 | -0.06 |
| Feels weak when upset | 1.38 | 0.65 | -0.07 |
| Subscale: Distracted |  |  |  |
| Work difficulty when upset | 1.77 | 0.78 | 0.02 |
| Difficulty thinking about other things when upset | 1.97 | 0.96 | 0.02 |
| Difficulty focusing on other things when upset | 2.03 | 0.97 | 0.12 |

*Note.* Unstandardized strength centrality, expected influence, and bridge expected influence values were examined in supplementary analyses. “Youth-Reported Depression Items” used the three youth self-reported depression items of the BPM instead of the parent-reported CBCL depression items at follow-up year 4. “All Items Included” included the two Difficulties with Emotion Regulation – Parent Report (DERS-P) items removed in the main network due to overlap with ADHD and depression symptoms. “Complete Cases” excluded participants who did not have ADHD, ER, and depression data (*n* = 4,460 total). “English-Speakers Only” excluded participants whose parents completed the DERS-P in Spanish (*n* = 9,916). “Item-Level Analyses” used the DERS-P items instead of subscale-level DERS-P as network nodes.

**Figure S6**

**Figure S7**

*Note.* Black squares indicate significant differences in bridge expected influence, *p* < .05. Gray squares indicate comparisons that are not significantly different, *p* > .05.

| **Table S4** |  |  |  |  |  |  |  |  |  |  |  |  |  |  |  |  |  |  |  |  |  |
| --- | --- | --- | --- | --- | --- | --- | --- | --- | --- | --- | --- | --- | --- | --- | --- | --- | --- | --- | --- | --- | --- |
| *Correlations between Nodes in the Network* | | | | | | | | | | | | | | | | | | | | | |
| Node | 1 | 2 | 3 | 4 | 5 | 6 | 7 | 8 | 9 | 10 | 11 | 12 | 13 | 14 | 15 | 16 | 17 | 18 | 19 | 20 | 21 |
| 1. Finish |  |  |  |  |  |  |  |  |  |  |  |  |  |  |  |  |  |  |  |  |  |
| 2. Concentr. | .69 |  |  |  |  |  |  |  |  |  |  |  |  |  |  |  |  |  |  |  |  |
| 3. Hyperact. | .52 | .72 |  |  |  |  |  |  |  |  |  |  |  |  |  |  |  |  |  |  |  |
| 4. Impulsive | .53 | .58 | .62 |  |  |  |  |  |  |  |  |  |  |  |  |  |  |  |  |  |  |
| 5. Inattent. | .68 | .85 | .68 | .63 |  |  |  |  |  |  |  |  |  |  |  |  |  |  |  |  |  |
| 6. Talks | .37 | .40 | .45 | .42 | .43 |  |  |  |  |  |  |  |  |  |  |  |  |  |  |  |  |
| 7. Too loud | .36 | .39 | .47 | .48 | .42 | .50 |  |  |  |  |  |  |  |  |  |  |  |  |  |  |  |
| 8. Enjoys little | .24 | .21 | .17 | .19 | .20 | .13 | .15 |  |  |  |  |  |  |  |  |  |  |  |  |  |  |
| 9. Cries lot | .11 | .08 | .07 | .09 | .07 | .10 | .12 | .21 |  |  |  |  |  |  |  |  |  |  |  |  |  |
| 10. Eats | .23 | .20 | .15 | .18 | .21 | .11 | .15 | .31 | .14 |  |  |  |  |  |  |  |  |  |  |  |  |
| 11. Worthle. | .24 | .22 | .17 | .21 | .24 | .14 | .17 | .39 | .27 | .27 |  |  |  |  |  |  |  |  |  |  |  |
| 12. Guilty | .12 | .10 | .10 | .10 | .12 | .13 | .10 | .23 | .23 | .14 | .36 |  |  |  |  |  |  |  |  |  |  |
| 13. Overtir. | .19 | .15 | .11 | .13 | .16 | .13 | .13 | .36 | .19 | .31 | .34 | .26 |  |  |  |  |  |  |  |  |  |
| 14. Less sleep | .17 | .16 | .17 | .17 | .16 | .12 | .14 | .23 | .10 | .22 | .19 | .12 | .19 |  |  |  |  |  |  |  |  |
| 15. More sleep | .13 | .11 | .09 | .09 | .11 | .10 | .12 | .26 | .16 | .22 | .20 | .13 | .41 | .10 |  |  |  |  |  |  |  |
| Node | 1 | 2 | 3 | 4 | 5 | 6 | 7 | 8 | 9 | 10 | 11 | 12 | 13 | 14 | 15 | 16 | 17 | 18 | 19 | 20 | 21 |
| 16. Trouble sleep | .20 | .18 | .16 | .17 | .19 | .14 | .13 | .27 | .15 | .30 | .29 | .19 | .36 | .45 | .24 |  |  |  |  |  |  |
| 17. Underactive | .24 | .19 | .12 | .15 | .21 | .14 | .13 | .38 | .15 | .32 | .33 | .18 | .47 | .20 | .31 | .31 |  |  |  |  |  |
| 18. Unhapp. | .21 | .16 | .10 | .14 | .16 | .11 | .13 | .48 | .31 | .31 | .51 | .31 | .39 | .21 | .26 | .37 | .41 |  |  |  |  |
| 19. Attuned | .22 | .20 | .16 | .20 | .20 | .04 | .12 | .17 | .03 | .16 | .15 | .06 | .12 | .07 | .09 | .10 | .12 | .13 |  |  |  |
| 20. Catastro. | .34 | .34 | .33 | .39 | .32 | .22 | .28 | .27 | .19 | .23 | .32 | .22 | .21 | .16 | .15 | .19 | .19 | .28 | .32 |  |  |
| 21. Neg. Secondary | .19 | .18 | .16 | .16 | .17 | .13 | .13 | .17 | .13 | .11 | .25 | .23 | .16 | .11 | .12 | .11 | .13 | .19 | .11 | .58 |  |
| 22. Distract | .40 | .38 | .33 | .38 | .40 | .21 | .26 | .24 | .15 | .24 | .31 | .20 | .19 | .16 | .13 | .21 | .21 | .27 | .22 | .71 | .49 |
|  |  |  |  |  |  |  |  |  |  |  |  |  |  |  |  |  |  |  |  |  |  |
| *Note. n* = 11,866. Correlations are pairwise comparisons. All correlations are significant, *p* < .05 | | | | | | | | | | | | | | | | | | | | | |
